# Nickel Sensitivity in Patients With Irritable Bowel Syndrome

**DOI:** 10.7759/cureus.45196

**Published:** 2023-09-13

**Authors:** Süleyman Coşgun, Umut Polat, Muhammed Kaya, Mesut Sezikli

**Affiliations:** 1 Internal Medicine, Gastroenterology, Kütahya University of Health Sciences, Kütahya, TUR; 2 Internal Medicine, Emet Dr. Fazıl Doğan State Hospital, Kütahya, TUR; 3 Internal Medicine, Hitit University Faculty of Medicine, Çorum, TUR; 4 Gastroenterology, Hitit University Faculty of Medicine, Çorum, TUR

**Keywords:** nickel, contact dermatitis nickel, irritable bowel syndrome, contact allergen, nickel sensitivity

## Abstract

Aim: Nickel (Ni) is the most common contact allergen in the population. We aimed to investigate whether there is a relationship between Ni sensitivity and irritable bowel syndrome (IBS) in our study.

Materials and methods: This study was conducted in 50 patients with IBS who were followed up between March 2018 and July 2018, and 40 healthy people as a control group in a single center with a dermatology department. European standard contact allergen series Ni preparate and corticosteroid pomace were applied to the back region of the study subjects. The evaluation was performed at 48, 72, and 96 hours according to the scheme proposed by the International Contact Dermatitis Research Group (ICDRG). Those who reacted at 72 hours were considered to have Ni allergy.

Results: The female/male ratio was 24/26 and 18/22 in the patient and control groups, respectively. The mean ages of the patient and control groups were 42.82 (±10.65) and 39.77 (±11.21) years, respectively. Ni sensitivity was present in 40% of the patient group and 17.5% of the control group (p=0.03).

Conclusion: We believe that the presence of Ni sensitivity is important in the pathogenesis of IBS disease. In our study, Ni sensitivity was found to be quite high in IBS patients compared to the normal population.

## Introduction

Irritable bowel syndrome (IBS) is a gastrointestinal disorder characterized by alterations in bowel habits and involves chronic abdominal pain without any organic cause. IBS, which is the most commonly diagnosed gastrointestinal disorder, has variations geographically and comprises 10% of all attendances with gastroenterologists [1]. While the prevalence of IBS was 10-15% in North America, it was 11.5% in Europe [2-3]. The prevalence in Asian countries (between 0.8% and 14%) is generally lower than in European countries [4-5].

Several symptom-based criteria were recommended to standardize the diagnosis of IBS due to the lack of biological markers. The Rome IV criteria is the most used among the criteria. The presence of two of the following three criteria in a patient, including the presence of recurrent abdominal pain at least one day per week over at least six months, establishes the diagnosis of IBS: 1) related to defecation (relief), 2) involving changes in the frequency of defecation, and 3) changes of the appearance of feces [6].

Nickel (Ni) is one of the most common causes of contact allergy in the community [7-8]. Ni, a metal, can be incorporated into chemical compounds and alloys. People are often exposed to Ni through the skin. It is also taken into the body by breathing, drinking water, and consuming some foods [9]. Only 1 to 10% of dietary Ni is absorbed [10].

It was found that not only contact but also the oral administration of Ni sulfate (600-5,600 μg in a single dose) may cause eczema [11]. There are studies suggesting that Ni that induces contact dermatitis may also be associated with gastrointestinal diseases [12]. However, there are very few studies examining the relationship between Ni and IBS [13]. If such a coexistence exists, it is thought that the symptoms of patients can be improved and the cost of treatment may be reduced after a Ni-poor diet. In this study, we aimed to investigate whether there is a relationship between IBS and Ni sensitivity.

## Materials and methods

This study was conducted with 50 patients over the age of 18 and 40 healthy volunteers. Between March and July 2018, 50 patients who were diagnosed with IBS according to the Rome IV criteria, followed up in the gastroenterology clinic, who did not use any systemic medication and have dietary restrictions, were included in the study. Forty healthy volunteers (most of the patient’s healthy partners), who were on a similar diet, had similar ages, and had no skin or systemic disease, were also included in the study. Informed consent was obtained from all individual participants included in the study.

Fifty people with IBS diagnosis who participated in the study were not differentiated in terms of constipation-dominant IBS, diarrhea-dominant IBS, and pain-dominant IBS. In our study, the patients had Ni used in the European standard contact allergen series (Chemotechnique diagnostic, European baseline S-1000) and corticosteroid pomade was applied as control in the back region. The evaluation was done according to the scheme proposed by the International Contact Dermatitis Research Group (ICDRG) after 48, 72, and 96 hours, and the subjects with positive and negative reactions were recorded. The detection of Ni sensitivity was evaluated by a dermatologist.

In our study, the subjects who used topical corticosteroids for one week before; volunteers using systemic corticosteroids, immunosuppressive, and cytotoxic treatment for at least three weeks before; subjects using nonsteroidal anti-inflammatory and antihistaminic drugs, and pregnant women were excluded. Also, individuals who wore jewelry containing Ni in the last month were excluded from the study. Subjects were tested for Ni susceptibility only.

The data about people included in the patient and control groups like age, sex, occupation, hobbies, family history, duration of disease, and history of allergic sensitivity were investigated and recorded on the forms prepared. The patients were warned not to take any baths, to avoid activities that would cause excessive sweating, and not to use drugs that would cause mistaken evaluations of the test. Reactions that were positive at the 48th hour and negative or decreased at 72 hours were accepted as irritants. The study was approved by the local ethics committee (Kocaeli Üniversitesi Klinik Araştırmalar Etik Kurulu 2018/93, 20/02/2018). For the statistical evaluation of the data, the Statistical Package for Social Sciences (SPSS) 17 package program (IBM Corp., Armonk, NY) was used. The patch test reactions of the two groups were compared with the chi-square test and a p-value less than 0.05 was considered significant.

## Results

There were 50 subjects in the patient group and 40 in the control group. The female/male ratio was 24/26 and 18/22 in the patient and control groups, respectively. The mean age of the patient and control groups was 42.82 (±10.66) and 39.77 (±11.21) years, respectively. Both groups were statistically similar in terms of age and gender distribution (p>0.005) (Table 1).

**Table 1:** Demographic characteristics of patients and control groups and Ni sensitivity rates Ni, nickel

Demographic characteristics	Patient	Control	p
Age (mean±SS)	42.82 (±10.65735 )	39.77 (±11.21)	0.19
Gender (f/m)	24/26	18/22	0.77
Ni sensitivity (%)	40	17.5	0.03

In our study, positive results were compared in terms of gender distribution in the patient and control groups. Of those with positive Ni sensitivity, 55% (11/20) and 71.4% (5/7) were female in the patient and control groups, respectively. The difference was not statistically significant (p=0.57). When the distribution of Ni sensitivity according to gender was examined in patients with IBS, the rate of positivity was found to be 45.8% in female patients and 34.6% in male patients, but no significant difference was found between genders. 

Ni sensitivity of the patient group was 40%, and the sensitivity of the control group was 17.5%. The difference between them was statistically significant (p=0.03).

When men and women were evaluated separately, Ni sensitivity was positive in 11 women (45.8%) in the patient group and five women (27.7%) in the control group. But Ni sensitivity was positive in nine men (34.6%) in the patient group, and two men (9.09%) in the control group (Table 2 and Table 3).

**Table 2:** Ni sensitivity rates between patient and control groups in women Ni, nickel

Women	Negative	Positive	Total
Patient	13	11	24
Control	13	5	18
Total	26	16	42

**Table 3:** Ni sensitivity rates between patient and control groups in men Ni, nickel

Men	Negative	Positive	Total
Patient	17	9	26
Control	20	2	22
Total	37	11	48

There was no significant difference between males and females in terms of Ni sensitivity. There was no difference between women of the patient group and the control group in terms of Ni sensitivity. However, there was a higher rate of Ni sensitivity in men in the patient group than in the control group, which was significant (p=0.03).

No volunteer had allergic reactions due to corticosteroids. Irritant reactions were not observed in both groups. However, mild erythema that occurred in patients was evaluated as suspicious and negative.

## Discussion

Ni, which can be found everywhere in the environment, is an element that can be taken into the body through diet, breathing, touching, and others. A recent study reported that IBS-like symptoms can be seen in people eating Ni-rich foods [14]. In a study by Kageyama et al., a susceptibility study involving Ni and various metals in IBS patients, a significant increase in sensitivity was reported compared to the healthy control [15].

Ni allergy is the most common metal allergy. In parallel to that, a study by Lu et al. also reported that the most common metal allergen in patients who underwent a patch test was Ni [16]. In our study, the results for the control group were consistent with the literature.

In a study published by Rizzi et al. in 2017, Ni susceptibility was higher in IBS patients compared to the healthy population, and symptoms improved with a low Ni diet. These findings lead researchers to develop hypotheses related to pathophysiological mechanisms linking IBS with Ni susceptibility [13].

In our study, the sensitivity of Ni in patients with IBS was significantly higher than in healthy individuals, and this result is similar to the data of Rizzi et al. The results of our study, which included 50 subjects, support the study by Rizzi et al., which included 20 patients. There were more IBS cases in women than men. Factors like women’s more frequent referral to the doctor as a result of cultural differences, being more sensitive to psychological stress, and differences in sexes in serotonin synthesis in the central nervous system may cause IBS to be seen more frequently in women than men [17]. Ni sensitization affected men due to more occupational exposure in the past. However, Ni sensitivity has begun to be seen more in women, especially at younger ages, because of the increasing use of imitation jewelry [18].

It is known that dietary Ni may be responsible for gastrointestinal symptoms that mimic the clinical features of IBS [19]. Worsening postprandial symptoms and lack of tolerance to foods are common in IBS disease [20]. The British Association of Dietitians stated that diet and lifestyle changes should be routinely offered to IBS patients [21]. In another study by Borghini et al., it is stated that a low-Ni diet reduces IBS-like complaints in patients with a diagnosis of endometriosis [22]. The correction of symptoms in IBS usually takes a long time. In order to achieve a significant improvement, a period of six months or longer is often required. 

Limitations

Although Ni is introduced into the diet at an earlier age in the general population, the age at diagnosis of IBS is later, and it was not investigated in our study. Also, a limitation is to compare the Ni sensitivity in the general population with our study in this small number of patients.

## Conclusions

As a result of this study, it is thought that dietary Ni intake may have a role in the etiology of IBS. We found Ni sensitivity to be significant in our study. There are still few studies about this subject, and new studies involving more patients are needed.
